# The impacts on the economy, health, and environment resulting from tobacco cultivation: A cross-sectional survey of tobacco farmer perspectives in Thailand

**DOI:** 10.18332/tid/204301

**Published:** 2025-05-24

**Authors:** Chakkraphan Phetphum, Raphael Lencucha

**Affiliations:** 1 Department of Community Health, Faculty of Public Health, Naresuan University, Phitsanulok, Thailand; 2 Tobacco Control Research Unit, Naresuan University, Phitsanulok, Thailand; 3 School of Physical and Occupational Therapy, Faculty of Medicine and Health Sciences, McGill University, Montreal, Canada

**Keywords:** tobacco farmers, tobacco cultivation, economic impact, health impact, environmental impact

## Abstract

**INTRODUCTION:**

Tobacco cultivation is associated with financial instability, health risks, and environmental degradation. While Thailand has made progress in tobacco control, challenges remain in supporting farmers with sustainable alternatives. This study examined the perceived economic, health, and environmental impacts of tobacco cultivation among Thai tobacco farmers.

**METHODS:**

A cross-sectional survey was conducted from October 2021 to January 2022 in Chiang Mai, Phrae, and Sukhothai, the major tobacco-growing provinces in Thailand. A total of 1505 tobacco farmers completed self-administered questionnaires. The instrument measured perceived impacts on a 3-point Likert scale (low to high). Frequencies and proportions for descriptive statistics are reported along with adjusted odds ratios and 95% confidence intervals for logistic regression models.

**RESULTS:**

Economic impacts were most frequently reported (43.7%), particularly increased debt (47.6%) and income loss (43.5%). Health impacts (31.6%) included symptoms of Green Tobacco Sickness (47.2%) and reduced work capacity (29.9%). Environmental concerns (14.4%) included pesticide contamination (10.8%) and degradation of soil and water resources (10.6%). Higher economic impact was associated with cultivating Virginia tobacco (AOR=6.51; 95% CI: 4.90–8.63), higher level of education (AOR=1.39; 95% CI: 1.01–1.92), contract farming (AOR=1.27; 95% CI: 0.99–1.63), and farming experience (AOR=1.00; 95% CI: 0.99–1.01). Health impact was associated with age (AOR=1.04; 95% CI: 1.03–1.05), land rental (AOR=0.75; 95% CI: 0.58–0.98), female gender (AOR=0.74; 95% CI: 0.58–0.94), and Virginia cultivation (AOR=0.32; 95% CI: 0.23–0.44). Environmental impact was linked to labor hiring (AOR=2.68; 95% CI: 1.41–5.07) and land rental (AOR=0.56; 95% CI: 0.39–0.79).

**CONCLUSIONS:**

Thai tobacco farmers face significant economic, health, and environmental burdens. Policy interventions should promote sustainable alternatives to mitigate these impacts.

## INTRODUCTION

Tobacco cultivation has historically been viewed as a lucrative source of revenue for governments and a stable livelihood for farmers, particularly small-scale farmers^1,2^. Major global tobacco companies suggest that tobacco farmers in various countries such as Pakistan, Argentina, Malawi, Turkey, and the Philippines have sufficient income from tobacco farming as a livelihood^1^. Farmers cite that they are attracted to tobacco growing by this promise of a sustainable livelihood^3,4^. However, this promise is often confronted by the reality of limited profits, debt, significant health risks, and harmful environmental impacts that arise from tobacco cultivation^5,6^. Studies continue to find that smallholder farmers rarely generate sufficient income to meet household needs and often find themselves in perpetual debt^7-11^.

The health consequences of tobacco growing are extensive. Tobacco farm workers have substantial exposure to hazardous levels of nicotine. This exposure to nicotine is linked to acute nicotine toxicity caused by dermal absorption through skin contact with tobacco leaves, leading to a condition known as Green Tobacco Sickness (GTS), which results in physical symptoms such as vomiting, nausea, headache, and dizziness^12,13^. A study by Ali et al.^14^ compared paddy and tobacco farmers in Bangladesh and found that tobacco farmers had far higher rates of these symptoms. The labor-intensive tobacco cultivation and production tasks commonly rely on family labor and involve repetitive physical movements throughout the day. This significantly increases the risk of developing musculoskeletal disorders (MSDs), both acute and chronic, due to the repetitive postures and strenuous physical effort involved^15,16^. Therefore, tobacco cultivation is a major contributor to illnesses that incapacitate individuals from working and necessitate hospitalization, leading to significant healthcare expenses. This encompasses medical expenses, medication, hospital stays, transportation, other indirect costs, lost workdays, and higher healthcare expenses compared to non-tobacco farmers^9^.

Environmental impacts resulting from tobacco cultivation and production are another significant issue extensively discussed in research. Tobacco cultivation has been found to affect soil micronutrient content and increase soil acidity^17^. Studies have detected the presence of toxic and potentially toxic elements (PTEs) such as cadmium (Cd) and arsenic (As) in the tobacco fields and soil, raising concerns about the potential exposure of tobacco farmers to levels beyond safety limits^18,19^. Research has confirmed the presence of organochlorine pesticides such as hexachlorocyclohexane (HCHs) and heptachlor, which are widely used in tobacco cultivation and represent significant environmental contamination. These pesticides are commonly found in the main river systems of tobacco-producing countries, indicating substantial environmental risks associated with tobacco cultivation^20^. Research with tobacco farmers in Thailand finds that growers experience a prevalence of Green Tobacco Sickness (GTS) at 22.6%^21^. A majority of them have a history of direct contact with tobacco leaves and report experiencing at least one of the following symptoms: nausea, vomiting, headache, and dizziness. Furthermore, some exhibit abnormal symptoms related to musculoskeletal disorders (MSDs), predominantly affecting the lower back, knees, shoulders, wrists, and hips^16^. Moreover, it has been observed that nearly all tobacco farmers have household debts^22^ and a generally poor overall quality of life^22^. Moreover, a broader assessment of tobacco cultivation and production practices in certain countries has shown that tobacco companies often adhere to low environmental standards. This includes poor practices in using chemical pesticides and fertilizers, as well as inadequate pollution prevention measures^23^.

Thailand is recognized as a regional leader in tobacco control^24^, yet tobacco cultivation and manufacturing remain significant^25^. The Thailand Tobacco Monopoly, established in 1939, was restructured into the Tobacco Authority of Thailand (TOAT) in 2018 to modernize operations. At its peak, TOAT had registered around 80000 tobacco farmers who supplied tobacco leaves under a licensing and quota system administered by the Ministry of Finance^26^. TOAT purchases tobacco leaves from farmers for both domestic and export cigarette production. In 2014, 30319 million cigarettes were sold domestically, declining to 18508 million in 2018, while annual exports ranged from 30 to 75 million cigarettes. This decline is attributed to the rise in foreign brand market share (40.3%)^26^. As a result, TOAT reduced tobacco leaf quotas, directly affecting farmers' income and contributing to a significant drop in domestic tobacco production. In 2022, in response to growing concerns about the livelihoods of tobacco farmers, a committee comprising ministries and civil society was established to provide financial assistance to affected farmers. However, comprehensive and sustainable long-term solutions for supporting these farmers remain limited^27^. According to Thailand’s 2016 FCTC implementation report, Articles 17 and 18 – concerning support for alternative livelihoods and environmental protection – showed the weakest progress^26^. As of 2022, approximately 16300 tobacco farmers remained registered with TOAT, reflecting both the continued reliance of rural households on tobacco and the urgent need for policy attention. This study examines tobacco cultivation’s perceived economic, health, and environmental impacts from the perspective of tobacco farmers and identifies contributing factors, providing timely evidence to inform future policy and farmer support strategies.

## METHODS

### Study design and ethics statement

This study applied a cross-sectional survey design. Data were collected from October 2021 to January 2022. The study was approved by the Ethics Committee in Human Research at Naresuan University (Project Number: P3-0087/2564), with ethical clearance granted on 24 April 2021.

### Setting and participants

This study surveyed tobacco farmers in the northern region of Thailand who met the following inclusion criteria: 1) they were either the head of the household or the person primarily responsible for growing tobacco within the household, 2) they held a valid license for growing Virginia or Burley tobacco varieties issued by the Tobacco Authority of Thailand (including both contract and independent farmers), 3) they had actively grown tobacco during the previous agricultural season, and 4) they provided informed consent to participate in the research study. Those unavailable during data collection were excluded from the survey.

The sampling framework for this study consists of tobacco farmers registered with the Thailand Tobacco Monopoly in three provinces designated as research areas. The sample was selected explicitly from Chiang Mai, Phrae, and Sukhothai provinces, which are recognized as the leading tobacco producers in the country^25^. The researchers calculated the sample size using the finite population proportion estimation formula with the given parameters: N=16300, p=0.226 (derived from the reported health impact rate of GTS disease, which is 22.6% as reported in Saleeon et al.^21^), δ=0.0226 (10% of p), α=0.05, and Z(0.975)=1.959964. When calculated according to the formula, the sample size was 1218 individuals. To account for non-response and potential errors due to incomplete questionnaire responses, an additional 25% of the calculated sample size was added. Thus, the target sample size was 1624 individuals.

Systematic Random Sampling was employed by establishing the sampling interval using the formula N(population size)/n(sample size), which resulted in an interval of 10.04. After determining the interval, a Simple Random Sampling approach was used through a lottery draw to select the first representative from the sequence numbers derived from the registration list of farmers at the Provincial Agriculture Office of Chiang Mai, Phrae, and Sukhothai. Respondents were systematically sampled in consecutive order, with an interval of 10 units from the previous sequence, until the total sample size of 1624 individuals was achieved.

### Measures

The data were collected through self-administered questionnaires developed by the primary author. The questionnaire consisted of four parts described below (Supplementary file). The economic, health, and environmental impact sections were based on questions developed through a literature review. Specifically, the economic domain addressed financial losses, increased debt, and a diminished quality of life due to insufficient income^5-11,22^. The health domain focused on the occurrence of Green Tobacco Sickness (GTS), illness requiring time off work or medical treatment, and musculoskeletal disorders^12-16,21^. The environmental domain covered degradation of soil and water resources, pesticide contamination, and pollution from tobacco production processes^17-20,23^. These themes informed the development of nine questions, three for each domain.

All questions were phrased positively, and a 3-point Likert scale was used, with options ranging from one (low or never occurred) to three (high). The total possible score ranged 3–9. The general characteristics of tobacco farmers were assessed using a combination of open-ended questions and checklist items, A total of 8 items, including gender (male, female), age (years), education level (no formal education or less than high school, higher than high school), experience in tobacco cultivation (years), cultivated land area for tobacco (rai), land ownership status for tobacco cultivation (landowner, leased land), labor utilization for tobacco cultivation (hired labor, family labor), and type of tobacco farmer (contractual tobacco farmer, independent tobacco farmer).

*Economic impact questions*

There were three specific questions to measure the perceptions of tobacco farmers over the past year regarding the economic impact: 1) ‘Have you encountered financial losses from tobacco cultivation?’, 2) ‘Have you accumulated increased debt due to tobacco cultivation?’ and 3) ‘Have you experienced a decline in your quality of life due to the income from tobacco cultivation not being sufficient to cover family living expenses?’.

*Health questions*

There were three specific questions to measure the perceptions of tobacco farmers over the past year regarding the health impact: 1) ‘Have you experienced symptoms of GTS (Green Tobacco Sickness) such as nausea, vomiting, headache, and dizziness upon contact with fresh tobacco leaves?’; 2) ‘Have you been sick to the extent that you couldn’t work or required hospital treatment due to tobacco cultivation?’; and 3) ‘Have you experienced abnormal muscle and bone conditions in the lower back, knees, shoulders, wrists, and hips that are predominantly a result of tobacco cultivation?’.

*Environment questions*

There were three specific questions to measure the perceptions of tobacco farmers over the past year regarding the environment impact: 1) ‘Have you encountered degradation of soil and water sources used for tobacco cultivation?’; 2) ‘Have the chemical pesticides used in your tobacco cultivation contaminated the environment?’; and 3) ‘Does your tobacco cultivation and production process contribute to environmental pollution, such as smoke from curing tobacco and the smell from drying tobacco?’.

Three experts assessed content validity using the Index of Item-Objective Congruence (IOC). They assessed the alignment between questionnaire items and the research’s operational definitions. Each expert rated each item: 1 (clearly measures objective), -1 (not measured), or 0 (unclear objective). The experts’ ratings were used to calculate IOC scores for each item. The results showed that IOC values for all questions ranged 0.67–1.00^28^. Furthermore, Cronbach’s α was calculated to assess questionnaire reliability. Additionally, a pilot test was conducted with 35 non-sample tobacco farmers in Phetchabun province. Cronbach’s α for each subscale were: economic impact (0.768), health impact (0.868), and environment impact (0.818).

Data were collected from October 2021 to January 2022. The lead author trained 30 data collectors, many familiar with the target sample group, covering research objectives, data collection protocol, participant compensation, and field data collection skills. Participants who provided informed consent then completed the anonymous questionnaire voluntarily. Data collectors then conducted face-to-face surveys by visiting households in the target sample group. Data collectors adhered to strict COVID-19 prevention measures while conducting face-to-face surveys. All data collectors were required to be fully vaccinated (3 doses) and undergo daily rapid antigen tests. During face-to-face surveys, data collectors maintained a 1 m distance and wore face masks. Researchers provided ongoing guidance and monitored progress through regular communication with data collectors via mobile phones.

After collecting questionnaires from data collectors, the researchers ensured confidentiality by using anonymous surveys. Only researchers accessed the responses. This process yielded 1505 completed questionnaires, resulting in a response rate of 92.67%.

### Statistical analysis

Data analysis was performed using SPSS version 17.0 (Chigoe, IL, USA). Descriptive statistics, frequencies, percentages, means and standard deviations, were used to summarize participant characteristics and impact variables. Binary logistic regression was applied to identify factors associated with perceived economic, health, and environmental impacts.

For each regression model, continuous independent variables were dichotomized using the sample mean as the cutoff, based on the normal distribution and close alignment between mean and median values. Univariate logistic regression was first conducted, and variables with p<0.05 were entered into the multivariable models using the Enter method to control for confounding. Results were reported as crude odds ratios (OR), adjusted odds ratios (AOR); 95% confidence intervals (CI), and p-values, with significance set at α=0.05.

Model fit was assessed using the Hosmer-Lemeshow goodness-of-fit test. All three models demonstrated acceptable fit, with p>0.05: economic impact model (χ^2^=15.45, df=8, p=0.051), health impact model (χ^2^=19.15, df=8, p=0.140), and environmental impact model (χ^2^=12.27, df=8, p=0.140), indicating no significant difference between observed and predicted values across risk deciles.

## RESULTS

### The general characteristics of tobacco farmers

The final sample consisted of 1505 tobacco farmers. Most of the sample was male, with an average age of 52.1 ± 10.5 years. Most of the participants had little to no formal education or education below the level of junior high school. Their average annual income from tobacco cultivation was approximately 155891 THB (1000 Thai Baht about US$30) per household. Farmers suggested a profit of 20–30% of total income, which would result in an estimated annual profit of 31178–46767 THB, which is well below the provincial minimum wages of 308–330 THB per day and even below the international poverty line of 29.2 THB per day^29^. However, it is important to note that the income and profits were not calculated in this study and were estimated by the farmers. The majority of the tobacco farmers had an average cultivation experience of 20.5 ± 11.5 years and cultivated an average land area of 6.5 ± 4.2 rai (1 rai = 1600 m^2^ or 0.4 acres) per family. They predominantly cultivated tobacco on their own land, with the prominent variety being Burley. The labor force for tobacco cultivation was largely composed of hired workers. Within the sample, 60.2% of the participants were contractual tobacco farmers, engaging in agreements with tobacco companies, while the remaining 39.8% were independent tobacco farmers (Table 1).

**Table 1:** Demographic characteristics of the study sample, overall and by economic impact, health impact, and environment impact, a cross-sectional survey, Thailand, 2022 (N=1505)

*Characteristics*	*Total* *n (%)*	*Negative economic impact* *n (%)*		*Negative health impact* *n (%)*		*Negative environmental impact* *n (%)*	
		*High*	*Low*	*High*	*Low*	*High*	*Low*
**Total,** n	1505	658	847	476	1029	222	1283
**Sex**	1498 (100)						
Male	803 (53.6)	341 (42.5)	462 (57.5)	289 (36.0)	514 (64.0)	117 (14.6)	686 (85.4)
Female	695 (46.4)	313 (45.0)	382 (55.0)	185 (26.6)	510 (73.4)	104 (15.0)	591 (85.0)
**Age** (years), mean ± SD (N=1504)	52.1 ± 10.5	53.1 ± 0.4	51.2 ± 0.4	54.4 ± 0.5	51.0 ± 0.3	52.7 ± 0.7	52.0 ± 0.3
**Education level**	1496 (100)						
No formal education/completed middle school	1220 (81.6)	513 (42.0)	707 (58.0)	427 (35.0)	793 (65.0)	163 (13.4)	1057 (86.6)
Higher than middle school	276 (19.4)	139 (50.4)	137 (49.6)	46 (16.7)	230 (65.0)	57 (20.7)	219 (79.3)
**Tobacco growing experience** (years), mean ± SD (N=1489)	20.5 ± 11.5	19.9 ± 0.5	21.0 ± 0.4	22.8 ± 0.6	19.5 ± 0.3	23.8 ± 0.9	20.0 ± 0.3
Size of tobacco farming land* (Rai), mean ± SD (N=1496)	6.5 ± 4.2	6.5 ± 0.2	6.5 ± 0.1	6.3 ± 0.1	6.6 ± 0.2	6.6 ± 0.1	6.2 ± 0.2
**Cultivated tobacco varieties**	1505 (100)						
Virginia	505 (33.5)	364 (72.1)	141 (27.9)	87 (17.2)	418 (82.8)	84 (16.6)	421 (83.4)
Burley	1000 (66.5)	294 (29.4)	706 (70.6)	389 (38.9)	611 (61.1)	138 (13.8)	862 (86.2)
**Tobacco land ownership**	1504 (100)						
Own land	1014 (67.4)	410 (40.4)	604 (59.6)	354 (34.9)	660 (65.1)	172 (17.0)	842 (83.0)
Rented land	490 (32.6)	248 (50.6)	242 (49.4)	122 (24.9)	368 (75.1)	50 (10.2)	440 (89.8)
**Labor utilization**	1495 (100)						
Family labor	210 (14.0)	81 (38.6)	129 (61.4)	84 (40.0)	126 (60.0)	11 (5.2)	199 (94.8)
Hired labor	1285 (86.0)	568 (44.2)	717 (55.8)	390 (30.4)	895 (69.6)	208 (16.2)	1077 (83.8)
**Types of farmers**	1496 (100)						
Contract farmer	900 (60.2)	469 (52.1)	431 (47.9)	235 (26.1)	665 (73.9)	150 (16.7)	750 (83.3)
Independent farmer	596 (39.8)	185 (31.0)	411 (69.0)	239 (40.1)	357 (59.9)	72 (12.1)	524 (87.9)

For each regression model, continuous variables were transformed into binary variables using the sample mean as the cutoff.

* Rai: 1600 m^2^ or 0.4 acres.

### The impact of growing tobacco

The questions asked farmers to identify the scale of negative impact that tobacco farming had on health, economic, and environmental domains. The results present the proportion of respondents who indicated a high, moderate, or low negative impact. It should be noted that low negative impact does not imply that tobacco farming was viewed to have a positive impact in the domain, but rather that the negative impact was viewed as low. Proportions of tobacco farmers indicating high-level impacts were 43.7% (economic), 31.6% (health), and 14.1% (environment).

**Figure 1:**
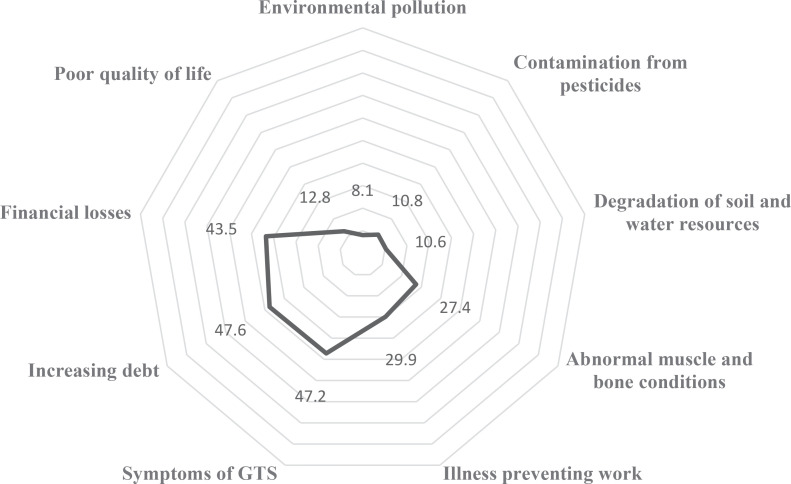
High-level impacts of tobacco cultivation on economy, health, and environment categorized by aspects, a cross-sectional survey, Thailand, 2022 (N=1505)

When considering the impacts individually. Figure 1 presents the perceived high-level impacts of tobacco cultivation across three key domains – economic, health, and environmental. Among the economic impacts, the most commonly reported issue was increasing debt, with 47.6% of farmers identifying it as a major concern. This was followed closely by financial losses from tobacco farming, reported by 43.5%, while 12.8% noted a decline in their overall quality of life due to insufficient income from tobacco.

Turning to health impacts, 47.2% of farmers reported experiencing Green Tobacco Sickness (GTS) – a condition associated with direct skin contact with wet tobacco leaves – making it the most common health complaint. Additionally, 29.9% of farmers indicated that tobacco-related illness had prevented them from working, and 27.4% reported musculoskeletal problems, likely caused by the repetitive and physically demanding nature of the work.

For environmental impacts, although less frequently perceived, 10.8% of respondents identified chemical contamination from pesticides as a serious issue. A further 10.6% noted soil and water degradation, and 8.1% cited environmental pollution, such as smoke from curing tobacco.

This visualization highlights the multidimensional burden tobacco cultivation places on farmers, with economic and health impacts dominating the landscape, and points to the need for greater awareness and investigation into environmental harm.

### Factors associated with the impact of tobacco cultivation

The analysis found four independent variables that explained the high economic impact scores when controlling for the influence of relevant variables (Table 2). The four variables are: cultivated tobacco Virginia (AOR=6.51; 95% CI: 4.90–8.63, p<0.001), education level higher than middle school (AOR=1.39; 95% CI: 1.01–1.92, p=0.045), Contract farmer status (AOR=1.27; 95% CI: 0.99–1.63, p=0.065), and experience (years) in tobacco cultivation (AOR=1.00; 95% CI: 0.99–1.01, p=0.513).

**Table 2:** Examining the associations between demographic characteristics, economic impact, health impact, and environment impact, a cross-sectional survey, Thailand, 2022 (N=1505)

*Variables*	*Economic impact*		*Health impact*		*Environment impact*	
	*OR (95% CI)*	*AOR (95% CI)*	*OR (95% CI)*	*AOR (95% CI)*	*OR (95% CI)*	*AOR (95% CI)*
Female ® Male	1.11 (0.91–1.36)		0.65 (0.52–0.81)*	0.74 (0.58–0.94)*	1.03 (0.78–1.37)	
Age (years)	1.02 (1.01–1.03)*		1.03 (1.02–1.04)*	1.04 (1.03–1.05)*	1.01 (0.99–1.02)	
Higher than middle school ® No formal education/completed middle school	1.40 (1.08–1.82)*	1.39 (1.01–1.92)*	0.37 (0.27–0.52)*		1.69 (1.21–2.36)*	2.09 (1.44–3.05)*
Tobacco growing experience (years)	0.99 (0.98–1.00)*	1.00 (0.99–1.01)	1.03 (1.02–1.04)*		1.69 (1.21–2.36)*	1.04 (1.02–1.05)*
Size of tobacco farming land (Rai)	1.00 (0.98–1.03)		0.98 (0.95–1.01)		0.98 (0.94–1.02)	
Cultivated tobacco –Virginia ® Burley	6.20 (4.89–7.86)*	6.51 (4.90–8.63)*	0.33 (0.25–0.43)*	0.32 (0.23–0.44)*	1.25 (0.93–1.67)	
Tobacco land – rented land ® Owned land	1.51 (1.22–1.88)*		0.62 (0.49–0.79)*	0.75 (0.58–0.98)*	0.56 (0.40–0.78)*	0.56 (0.39–0.79)*
Hired labor ® Family labor	1.26 (0.94–1.70)*		0.65 (0.48–0.88)*	0.67 (0.49–0.93)*	3.49 (1.87–6.53)*	2.68 (1.41–5.07)*
Contract farmer ® Independent farmer	2.42 (1.95–3.00)*	1.27 (0.99–1.63)	0.53 (0.42–0.66)*	0.79 (0.61–1.02)	1.46 (1.08–1.97)*	1.04 (0.96–1.82)

AOR: adjusted odds ratio; adjusted for variables in the table. Binary logistic regression was used to identify factors associated with each impact domain. Continuous variables were dichotomized using the sample mean. Variables with p<0.05 in univariate (crude) analysis were included in multivariable models using the Enter method. Model fit was assessed using the Hosmer-Lemeshow test: economic model (χ²=15.45, df=8, p=0.051), health model (χ²=19.15, df=8, p=0.140), and environmental model (χ²=12.27, df=8, p=0.140), all indicating acceptable model fit.

* p<0.05. ® Reference categories.

Furthermore, for the equation concerning the health impact of tobacco cultivation, there were a total of 6 jointly explanatory independent variables. These variables are: age (AOR=1.04; 95% CI: 1.03–1.05, p<0.001), contract farmer status (AOR=0.79; 95% CI: 0.61–1.02, p=0.073), Tobacco land rented by farmers (AOR=0.75; 95% CI: 0.58–0.98, p=0.034), female gender (AOR=0.74; 95% CI: 0.58–0.94, p=0.015), hiring of labor (AOR=0.67; 95% CI: 0.49–0.93, p<0.016), and cultivated tobacco Virginia (AOR=0.32; 95% CI: 0.23–0.44, p<0.001).

Lastly, for the equation concerning the environmental impact of tobacco cultivation, there were a total of 5 jointly explanatory independent variables. These variables are: hiring of labor (AOR=2.68; 95% CI: 1.41–5.07, p=0.003), education level higher than middle school (AOR=2.09; 95% CI: 1.44–3.05, p<0.001), contract farmer status (AOR=1.32; 95% CI: 0.96–1.82, p=0.088), experience (years) in tobacco cultivation (AOR=1.04; 95% CI: 1.02–1.05, p<0.001), and tobacco land rented by farmers (AOR=0.56; 95% CI: 0.39–0.79, p=0.001).

## DISCUSSION

This study provides insights into the perspectives of tobacco farmers in Thailand on the economic, health, and environmental features of tobacco farming. The most common impact identified by farmers in our sample is the economic impact, with a proportion of tobacco farmers indicating that they are facing high levels of negative economic consequences at 43.7%. The economic impact predominantly revolves around issues such as increasing debt, experiencing losses, and having a poor quality of life, all due to tobacco cultivation. This is in line with a large amount of empirical evidence that continues to reveal that most tobacco farmers are poor, have debts, and have a poor quality of life^7,10^. These problems are even more prevalent among contract tobacco farmers compared to independent tobacco farmers. This situation closely resembles what has been observed among tobacco farmers in other countries, such as Bangladesh, where contract farmers tend to have lower incomes from tobacco sales and higher expenses compared to independent tobacco farmers^11^. Research in other countries has pointed to the structural dynamics that attract farmers to contractual relationships with leaf buying companies, such as a lack of capital and a guaranteed market for the leaf at the end of the growing season. It is these same structural dynamics that render farmers vulnerable to inequities as part of the contractual relationship, such as high interest rates and lower prices than promised in the contract. The diversity of crops available to independent tobacco farmers in Thailand may allow them to have broader income sources^11^.

The health impact is another significant issue affecting the majority of tobacco farmers who directly perceive and experience the negative consequences on their health. Nearly one-third of tobacco farmers, almost 33%, indicate that they are significantly impacted by health issues resulting from tobacco cultivation. However, it is important to note that the proportion of individuals reporting these health impacts might be lower than the actual occurrence due to delays in recognizing symptoms associated with tobacco growing compared to laboratory measurements and the potential accumulation and latency period for health risks to manifest. The most prevalent health problem is related to Green Tobacco Sickness (GTS), with approximately 47.2% of tobacco farmers having experienced symptoms at least once after direct contact with fresh tobacco leaves. Symptoms include skin rashes, nausea, vomiting, headache, and dizziness. This aligns with existing research findings that confirm the high risk of skin absorption of nicotine in tobacco farmers, measurable through concentrations of cotinine in urine or saliva^12,21^. Cotinine levels in the body have been positively correlated with the likelihood of GTS occurrence^30^, particularly among those who handle tobacco leaves extensively, such as those involved in leaf-cutting, binding, and wet-leaf collection^30^. These findings are important to confirm that health issues persist for tobacco growers despite the extensive knowledge of the health harms. In the short-term, governments must recognize and work with farmers to mitigate these health risks.

The labor requirements of tobacco growing are extensive and are often much higher than those of other crops^6^. Around 30% of tobacco farmers noted the high level of experience with time off farming due to work-related illness, leading to not only work interruptions but also health-related costs such as medical expenses, medicines, hospitalization, transportation, and other incidental expenses. Additionally, these health problems contribute to higher costs compared to non-tobacco-farming agricultural workers^9^. Furthermore, approximately 27.4% of tobacco farmers experience abnormal muscle and skeletal conditions after tobacco cultivation. This is attributed to the physically demanding and repetitive nature of tobacco cultivation tasks, such as leaf handling, curing, and transportation^16^, which do not often involve advanced equipment or technology. The health impact of tobacco farming underscores the immediate health risks faced by these farmers, highlighting the importance of transitioning to alternative crops for their well-being and long-term health. Our findings also suggest differences in health and economic impact between the types of leaves grown. The process of Virginia tobacco curing, which requires the use of heat energy from wood-fired furnaces as a primary source, potentially leads to higher costs compared to Burley tobacco^31^ which utilizes air curing. The process of handling and drying and hanging fresh tobacco leaves in spaces close to residential dwellings may increase the risk of symptoms and diseases such as Green Tobacco Sickness (GTS).

Farmers’ perspectives on environmental impact requires further study. For example, it is possible that the small proportion of farmers who identified high environmental impact could stem from knowledge of environmental harms, risk prevention programs implemented by farmers to mitigate environmental harms or other factors. Our survey does not provide an indication of what factors shape the farmers responses. Given the extensive research indicating environmental harms of tobacco growing, there is reason to think that there is a need for greater education of these harms among farmers. However, as noted, further research is needed to confirm the factors that shape farmers perspectives on environmental harm.

This research provides a glimpse into the state of tobacco growing in Thailand. Thailand continues to be a global leader in efforts to strengthen tobacco control^24^. Jaroensathapornkul^32^ notes that the market for tobacco leaf grown in Thailand continues to shift and is impacted by global tobacco control efforts. Leaf grown in Thailand has experienced a significant decline in value on the global market suggesting that the economic hardships now being experienced by farmers will likely continue, as regions work to reduce consumption^32^. Governments like that of Thailand are poised to improve the well-being of tobacco farming households by systematically and collaboratively pursuing the World Health Organization’s Framework Convention on Tobacco Control (FCTC) under Article 17, which focuses on supporting economically viable alternative activities to tobacco cultivation. There is a body of learning emerging from countries such as China, Malawi, Kenya, and European countries where alternatives to tobacco are being rigorously pursued^33^.

### Strengths and limitations

This study offers valuable insights into the multifaceted impacts of tobacco cultivation from the perspectives of farmers themselves. By capturing farmers’ lived experiences, the study enhances understanding of how tobacco farming affects their economic security, health, and the environment. These insights are particularly relevant for informing future policy and support programs aimed at improving farmer livelihoods and promoting sustainable alternatives. Nonetheless, several limitations should be acknowledged. First, the data were collected during a period of economic disruption caused by the COVID-19 pandemic, which may affect the generalizability of findings to more typical economic contexts. It is possible that pandemic-related stressors amplified farmers’ perceptions of hardship. Future research conducted in more stable conditions would help confirm the robustness of these findings. Second, the cross-sectional nature of the study limits the ability to draw causal inferences. Although associations between farmer characteristics and perceived impacts were identified, the directionality of these relationships cannot be determined. Longitudinal studies are needed to assess how these impacts evolve over time, especially in response to changes in policy or market dynamics. Third, while this study explored subjective economic impacts, it did not collect quantitative data on household income, production costs, or profitability. Including these measures in future research would provide a more comprehensive understanding of the economic viability of tobacco cultivation and could help guide targeted interventions. Fourth, there is a possibility of residual confounding. Although multivariable logistic regression was employed to adjust for several key variables, unmeasured or imprecisely captured factors – such as intensity of pesticide use, informal labor conditions, or access to government support – may have influenced the results. Lastly, these findings are based on tobacco farmers in Thailand and may not be generalizable to other settings with different regulatory environments, climatic conditions, or farming systems. Contextual differences mean that similar studies are needed in other countries to identify both common patterns and context-specific challenges in tobacco farming. Despite these limitations, the study contributes significantly to the evidence base by documenting the real-world challenges faced by tobacco farmers and highlights the urgent need for structural support in transitioning to more sustainable agricultural practices.

## CONCLUSIONS

The direct perceived impacts of tobacco cultivation by the largest number of tobacco farmers are primarily economic problems. These include increasing debts due to tobacco cultivation, facing losses from tobacco cultivation, and having a poor quality of life. The secondary impacts are health-related problems, including experiencing symptoms of Green Tobacco Sickness (GTS) which causes illness from tobacco cultivation to the extent that one cannot work, as well as experiencing abnormal muscle and bone conditions. Subsequently, there are environmental problems, such as contamination from chemical pesticides, soil degradation, water resources depletion, and environmental pollution. This research provides substantial empirical evidence that contradicts the persuasive advertisements of tobacco companies. It suggests that the Thailand government should prioritize fair support policies to encourage tobacco farmers to successfully transition to cultivating alternative crops other than tobacco.

## Supplementary Material



## Data Availability

The data supporting this research are available from the authors on reasonable request.
