# Corrigendum: The impacts on the economy, health, and environment resulting from tobacco cultivation: A cross-sectional survey of tobacco farmer perspectives in Thailand

**DOI:** 10.18332/tid/210572

**Published:** 2025-09-19

**Authors:** Chakkraphan Phetphum, Raphael Lencucha

**Affiliations:** 1 Department of Community Health, Faculty of Public Health, Naresuan University, Phitsanulok, Thailand; 2 Tobacco Control Research Unit, Naresuan University, Phitsanulok, Thailand; 3 School of Physical and Occupational Therapy, Faculty of Medicine and Health Sciences, McGill University, Montreal, Canada

**Keywords:** tobacco farmers, tobacco cultivation, economic impact, health impact, environmental impact

Corrigendum on:

The impacts on the economy, health, and environment resulting from tobacco cultivation: A cross-sectional survey of tobacco farmer perspectives in Thailand By Chakkraphan Phetphum, Raphael Lencucha

Tobacco Induced Diseases, Volume 23, Issue May, Pages 1–11

Publish date: 24 May 2025

DOI: https://doi.org/10.18332/tid/204301

In the originally published version of the article, the AUTHORS’ CONTRIBUTIONS statement was not provided. The statement is now added as follows:

‘AUTHORS’ CONTRIBUTIONS

CP: research concept and design, collection and/or assembly of data, data analysis and interpretation. Both authors: writing the article, critical revision of the article. The authors read and approved the final version of the manuscript.’

The mentioned changes have also been completed online.

