# Supplementary material for: The impacts on the economy, health, and environment resulting from tobacco cultivation: A cross-sectional survey of tobacco farmer perspectives in Thailand
Source: Tob Induc Dis. 2025 May 24;23:10.18332/tid/204301. doi: 10.18332/tid/204301 (PMC12103073; doi:10.18332/tid/204301)
Supplement: Supplementary file 1 [file TID-23-70-s1.pdf]

Province no...............
ID no……………..

Questionnaire for tobacco farmers

This questionnaire aims to collect data on the economic, health, and environmental impacts of tobacco cultivation **over the past year** among tobacco farmers in Thailand.

This survey focuses on the ethical issues of human research. The researchers assure you that the information we receive from you will remain confidential, and will only be presented in an overview, which will not affect anything. In any case, you are free to refuse to provide information or stop answering the questionnaire anytime without any consequences.

This questionnaire consists of one page, divided into 4 parts as follows:
Part 1: General information (8 items)
Part 2: Health Questions (3 items)
Part 3: Economic Questions (3 items)
Part 4: Environment Questions (3 items)

We would like to ask for your cooperation in responding to this questionnaire as accurately and completely as possible.

The researchers would like to thank all of you for your cooperation at this opportunity.

Instructions: Please mark / in the box ☐ or fill in the blanks to match the actual situation.

Part 1 General Information.

1. Sex ☐ Male ☐ Female
2. Age ………………years
3. Educational level ☐ No formal education or less than high school ☐ Higher than high
4. Experience in Tobacco Cultivation ………………years
5. Cultivated Land Area for Tobacco ………………Rai
6. Land Ownership Status for Tobacco Cultivation ☐ Landowner ☐ Leased land
7. Labor Utilization for Tobacco Cultivation ☐ Hired labor ☐ Family labor
8. Type of Tobacco Farmer ☐ Contractual tobacco farmer ☐Independent tobacco farmer

**Part 2: Health Questions**	**Low or Never Occurred**	**Moderate**	**High**
1) Have you experienced symptoms of GTS (Green Tobacco Sickness) such as nausea, vomiting, headache, and dizziness upon contact with fresh tobacco leaves?			
2) Have you been sick to the extent that you couldn't work or required hospital treatment due to tobacco cultivation?			
3) Have you experienced abnormal muscle and bone conditions in the lower back, knees, shoulders, wrists, and hips that are predominantly a result of tobacco cultivation?			
**Part 3: Economic Questions**			
1) Have you encountered financial losses from tobacco cultivation?			
2) Have you accumulated increased debt due to tobacco cultivation?			
3) Have you experienced a decline in your quality of life due to the income from tobacco			

cultivation not being sufficient to cover family living expenses?			
**Part 4: Environment Questions**			
1) Have you encountered degradation of soil and water sources used for tobacco cultivation?			
2) Have the chemical pesticides used in your tobacco cultivation contaminated the environment?			
3) Does your tobacco cultivation and production process contribute to environmental pollution, such as smoke from curing tobacco and the smell from drying tobacco?			

© 2025 Phetphum C. and Lencucha R.
